# Farmers’ perceptions and attitudes towards the development of the sheep and goat sector in the Greek region of Evros

**DOI:** 10.1186/s40064-016-2811-3

**Published:** 2016-07-19

**Authors:** Stamatis Aggelopoulos, Christos Karelakis, Georgios Tsantopoulos, Alexandra Pavloudi, Paschalis Seitanis

**Affiliations:** Department of Agricultural Development and Agribusiness Management, Alexander Technology Educational Institute of Thessaloniki, Sindos, 57400 Thessaloníki, Greece; Department of Agricultural Development, Democritus University of Thrace, Pantazidou 193, 68200 Orestiada, Greece; Department of Forestry and Management of the Environment and Natural Resources, Democritus University of Thrace, Pantazidou 193, 68200 Orestiada, Greece

**Keywords:** Farming practices, Farmer attitudes, Farmers’ opinion, Sustainable development, Evros

## Abstract

The present study endeavors to investigate the attitudes, opinions and perceptions of livestock farmers regarding the main problems they face and confine the development of livestock in the Greek region of Evros. Primary data were collected through a quantitative survey (structured questionnaire) with livestock farmers in the region. The analysis of the survey data was carried out with the use of descriptive statistics, Friedman’s test and factor analysis. The results indicate that strategies for livestock development in the region should take into account the great dissatisfaction that exists towards public authorities and the level of satisfaction as regards the non-governmental bodies. Furthermore, the economic objectives are of primary importance for livestock farmers, whereas they stress their interest in training and know-how. Particularly on issues pertaining to dairy livestock management, improving sanitation conditions and enhancing the quality, digestibility and production of animal feed.

## Background

Livestock farming consists a key player in the economy of a large number of countries and until recently, it was practiced in a traditional manner. Nevertheless, large-scale investments have been observed in this sector and its structure has changed from nomadic to keeping animals in a lairage, characterized also by extensive mechanization in its main operations (Karelakis et al. 2013). The development of livestock farming does not only involve issues related to lairage facilities, equipment and the genetic improvement of livestock, but also concerns farmers’ attitudes on better livestock treatment practices and their knowledge on farming management issues and zootechnics (Te Velde et al. 2002; Vanhonacker et al. 2008; Dwyer 2009; Karelakis et al. 2013). Hence, a potential conflict may exist between seeking profitability and good animal health in livestock farming systems (Stott et al. 2005).

Clearly, livestock welfare is a value-laden concept and animal welfare science cannot be practiced independently of questions linked to values and ethics. Farmers should choose their production plan on the grounds of their fundamental objectives and their decisions should be based on their impact on the various parties (Jensen and Sørensen 1999; Whittemore 1995). If farmers obtain the necessary information on how their production practices affect the interests of other parties, then an ethical accounting system can be beneficial. Accordingly, farmers will gain new insights into their farms’ operations and the guidelines they should follow to assess the overall practices on their farm, if they want to act in accordance with ethical concerns. It has been noted that when sound management practices are not applied, livestock farming results in the downgrading of grazing areas, the pollution of water resources and the loss of biodiversity. On the contrary, good livestock practices may positively affect natural resources by improving soil quality and biodiversity. Where possible, these economic and environmental concerns should be promoted through attractive scenarios, and the relevant policies and technologies that will contribute to this aim, should be defined (Boyazoglu 2002).

The prefecture of Evros, which is the area under study, experiences one of the worst trials in its history as whole herds of sheep (primarily) have been decimated by sheep pox and bluetongue, due to the lack of effective means and support structures to confront this problem. According to the Regional Veterinary Directorate, in the early stages of progression of smallpox disease, the total number of slaughtered animals was 18,158 in 117 farms and about 70,000 the compensated animals. This issue, along with the absence of any national policy for livestock farming, have led the sector and the overall growth of the region to a marginal point. The sustainability of livestock farms is essential for the future of the sector and can become the driving force for further regional development. Despite the large number of extant literature regarding the development of livestock farming in Greece in recent years (Fousekis et al. 2001; Aggelopoulos et al. 2010; Galanopoulos et al. 2011; Aggelopoulos et al. 2013; Karelakis et al. 2013; Pavloudi et al. 2013), and Manousidis et al. (2016) who studied the factors influencing the dietary preferences of goats in the study area, international scholars have not discussed the ethical questions that arise from the loss of a large number of animals.

Accordingly, the objective of the present study is to define certain development policy measures for the sheep and goat farming sector, based on farmers’ attitudes and perceptions. Specifically, the research endeavors to examine the views of livestock farmers on issues related to the future growth of the sector. The various factors that will emerge from the study may consist a platform for policy-makers to contribute to the sector’s development in this region. The remainder of the paper includes a description of sheep and goat farming in Greece in the next section, whereas the third section offer a general picture of the area under study. The fourth section describes the materials and methods used in the current study along with the main results in the fifth section. Finally, the last section concludes.

## Characteristics of sheep and goat farming in Greece

Greece has a long tradition in livestock farming, mainly sheep and goat (Pavloudi et al. 2013) and many studies have assessed the economics and overall sustainability of the sheep and goat farming sector, along with the potential for further improving its competitive profile. Aggelopoulos et al. (2010), argued that the key problem affecting the competitiveness of the sector involves the high production. Theocharopoulos et al. (2007) analyzed the input expenses and specified the technical efficiency of sheep farms. They determined the possibility of reducing production costs by improving the technical efficiency of farms, in order to deal with the elimination of subsidies within the framework of the CAP (Common Agricultural Policy) measures. Fousekis et al. (2001), defined the overall efficiency of sheep farms, whereas Galanopoulos et al. (2011), observed that nomadic sheep and goat farming, despite its decline, comprises a primary source of income for less favored and mountainous areas.

The livestock sector has followed a downward trend recently, due to a decrease in production, livestock and employment. Greece presents a deficit in most livestock farming products, and consequently has to import milk and meat. This negatively affects the country’s trade balance and the main reason is the increased production costs compared to other countries (Reziti 2015). In addition, the employment level is still at low despite the crucial role of livestock farming in the regional agricultural development and in preserving the social fabric in rural areas (MRDF 2011).

According to data from the Economic Accounts of Agriculture, in 2013 the ratio between plant and animal production as regards value (70/30) remained at the same levels as in the ‘80s. The contribution of Greek livestock farming to ΕU-28 is small (1.6 %), in contrast to its share in sheep and goat farming (12.8 %). As a whole, in the period 2011–2013, the value of animal production decreased by 4 %, while the value of plant production dropped by 9.5 %. In addition, in 2013, the value of animal production decreased by 1.3 %, i.e. at a slower rate than the value of plant production (−8.4 %). After studying the participation of the various sectors and animal products in the gross value of animal production, it is concluded that the most important animal product is milk (11.6 %) and the most active sector of livestock farming is sheep and goat farming (7.0 %).

## Study area

The prefecture of Evros, covers an area of 4242 km^2^ and is located in the north-eastern part of Greece, with a population of 147,947 people (HELSTAT 2016). Its inhabitants are mainly involved in the production of agricultural products, either individually or collectively through local cooperatives. The agricultural sector is primarily oriented to grain cultivation, whereas other crops include cotton, maize, alfalfa, sugar beet, sunflowers and various types of trees for wood production, fruit and other harvests. Other trees are cultivated for their leaves, such as mulberries, whose leaves are used for sericulture and the production of silk. The shift of the CAP orientation, from production subsidies to restructuring programs that require more dynamic initiatives and a systematic mobilization on a national and local level, has created obstacles for the future socio-economic status of the region. Yet, the prefecture’s strategic location offers the possibility for its products to be distributed to foreign markets. The development of relevant infrastructure and its connection to trans-European networks have made the prefecture a centre for transit trade to Northern Europe and the Balkans (Dimitriou et al. 2010).

Livestock farming is the second most important sector, as regards employment, in the prefecture, despite its noticeable decline trend. On the other hand, the farming of large animals has increased, quantitatively and qualitatively, mainly in the area of Feres and the Evros Delta; an area used for grazing a large number of cattle for meat production. The growth of agriculture and livestock farming is largely attributed to the various programs run by the European Union. According to the census of 2009, there are 1005 animal farms with 137,126 sheep and 805 holdings with 84,138 goats in Evros Prefecture.

The prefecture’s Gross Domestic Product (GDP) per capita accounts for €14.000 comprising the 15.1 % of the European equivalent and the 0.9 % of the total GDP in the country Dimitriou et al. (2010). Although the region’s economic activities are mainly centered on agricultural production, its share in employment being 28.3 %, the primary sector’s share in the prefecture’s GDP is lower than that of the two other sectors. Specifically, the primary sector accounts for 8.5 % of the GDP, the secondary sector for 17.6 % and the tertiary sector for 73.9 % (HELSTAT 2016).

## Methods

Primary data were collected through personal interviews with the livestock farmers (structured questionnaire) from June to November 2014. The survey instrument was created after taking into account the relevant literature and the present status of sheep and goat farming in the prefecture. Prior to the questionnaire distribution, the necessary tests were conducted to check content validity (Zikmund 2003), which examined the degree of comprehension, “acceptance”, and interpretation of the questionnaire. Preliminary testing was required in order to: avoid using unsuitable, biased, vague or duplicated questions, determine the order of the questions so that any possible distortion trends were prevented, to reduce the length of the questionnaire and avoid any indifference being shown by the interviewees. Accordingly, the questionnaire included questions that were related to the socio-economic characteristics of the farmers and of the farms, such as the business structure of the holding, whether it belongs to a cooperative, if the farmers practice any other profession besides livestock farming etc., and questions related to the farmers’ objectives and their livestock farm management practices.

The population under study comprised livestock farmers from the Evros prefecture. The respondents were selected with a simple random sampling from four out of the five municipalities in the prefecture. These were the municipalities of Alexandroupoli, Soufli, Didymoteicho and Orestiada (the municipality of Samothrace was not included). After reviewing the relevant literature (Damianou 1999; Kalamatianou 2000; Matis 2001) the sample size accounted to ninety sheep and goat farmers. The questionnaires were completed using personal interviews and the data processing was carried out through a series of multivariate methods that included the a-Crοnbach coefficient, the descriptive statistics, the Friedman’s non-parametric criterion, and the principal component analysis (PCA) (Hair et al. 1995).

## Results

The majority of the sheep and goat farmers were men (84.4 %), who had also the primary responsibility for the decisions and operation of the holding. They had a low educational level, since 63.3 % had only completed primary education, or not even that. Most of them were aged between 45 and 65 years old and approximately half of them had their farm for over 20 years; none of the farmers were aged under 25 years old. Most sheep and goat farms employed two persons (family members) for about 10 h daily. There were also farms with full-time or part-time workers, who were employed for about 11 h daily. Finally, the annual income for the majority of respondents ranged between 10,000 and 30,000 euros. Any differences were due to herd size, the efficient herd and products’ management, and the percentage of feedstuff grown on the farm. For 2011, the average rural income in the region accounted for €279.24 million compared to €4594.64 million (6.07 % of the country’s rural income) (HELSTAT 2016). Farmers also possessed an average of 200 sheep and around 182 goats; 14.4 % of the farmers owned over 350 animals.

About half of the farmers (46.7 %) were involved exclusively in livestock farming, while the other half were also involved in agriculture as a supplementary profession; there were only very few exceptions, involving farmers who also had other professions. Of the total number, 8.9 % were organic animal farms, while 60 % of the interviewees had legalized their lairage facilities, and several had initiated the process of legalization. They mainly used makeshift lairage facilities made of wood and metal sheeting; only 16.7 % had modern facilities. About 30 % used a milking pen for milking the animals, while the rest of the farmers argued that milking was done manually.

**Fig. 1:**
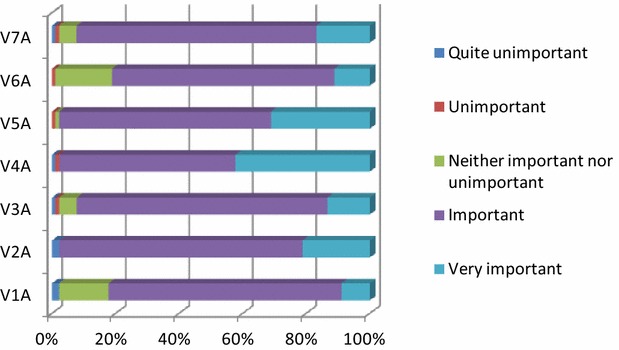
Percentage points depending on the significance of livestock farming objectives. V1A = soil maintenance and conservation; V2A = profit maximization; V3A = farm size increase; V4A = damage avoidance; V5A = capital increase; V6A = time for other activities; V7A = family participation in the farm

Regarding their potential objectives (Fig. 1), farmers considered quite important to maintain and conserve the soil, so that it can be used by future generations. Other important objectives included the profit maximization on an annual basis (given the available resources) and the increase in the farm’s size. The latter does not only involve an increase in livestock numbers, but also the use of technology and improving the lairage facilities.

Other significant objectives set by the sheep and goat farmers were to avoid any losses regarding the livestock, the animal feed and lairage facilities, the increase in capital to prevent abandonment and the sound farm management. A high percentage of sheep and goat farmers considered important to have time for other activities, while family participation in the farm was viewed as being equally important. In order to examine the potential existence of a statistical difference between the farmers’ views on the significance of the noted objectives, the Friedman’s statistical test was applied. According to the results, it was established that “damage avoidance” is the main goal of the farmers with a mean ranking of 4.84 (Ν = 90, χ^2^ = 82.893, df = 6, Asymp. Sig < 0.001).

The application of PCA highlighted two major factors that explain 61.57 % of the total variance (a-Cronbach = 0.723, ΚΜΟ = 0.653, χ^2^ = 227.765, df = 21, p < 0.001). The following Table 1 presents the loadings, which constitute the partial correlation coefficients of the seven variables with each of the two factors that emerged from the analysis.

**Table 1:** Factor loadings regarding the significance of farmers’ objectives

Factor loadings		
Variable	Following rotation	
Damage avoidance	*0.880*	−0.091
Profit maximization	*0.869*	0.085
Farm size increase	*0.804*	0.146
Soil maintenance and conservation	*0.761*	0.028
Time for other activities	−0.020	*0.774*
Family participation in farm	−0.026	*0.698*
Invested capital increase	0.410	*0.513*

The first factor comprises “damage avoidance”, “profit maximization”, “farm size increase” and “soil maintenance and conservation” and is potentially an expression of the farmers’ “economic and sustainable objectives”. It is clear that the greatest impact on the formulation of the first factor is related to damage avoidance, while “soil maintenance and conservation” display the least impact. This can be interpreted in relation to the sustainability problems that sheep and goat farms are confronting. This is why these farms are more concentrated to avoid any additional costs rather than improving their economic results. Finally, the second factor specifies the availability of time for other activities as an objective along with the valorization of family labor in conjunction with an increase in farm investments. The figures show that the exploitation of human resources plays the greatest role in the formulation of the second factor. The investments realized in these holdings aim at valorizing family labor in a better and more productive way.

**Fig. 2:**
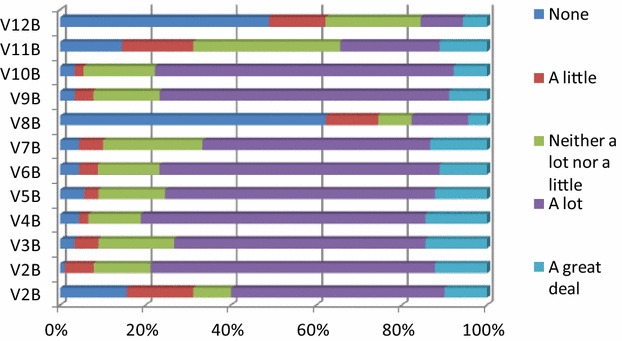
Percentage points regarding proposed herd management practices. V2B = use of improved breeds; V2B = improved sanitation conditions; V3B = management of pregnant animals; V4B = management of dairy animals; V5B = management of replacement stock; V6B = rate of stock replacement; V7B = lairage systems and well-being; V8B = management practices for organic production; V9B = use of rational feeding systems; V10B = quality, digestibility and production of animal feed; V11B = recording, analysis and monitoring of production, animal health and reproduction; V12B = electronic milking systems and their management

As regards knowledge or training on farm management practices, a large number sheep and goat farmers wish to improve their knowledge regarding lairage facilities, while 62.2 % of them show no interest in management practices for organic production. Enhancing their knowledge on the use of rational feeding systems and on the quality, digestibility and production of animal feed, are also significant for the majority of farmers. The recording, analysis and monitoring of production and of the health and reproduction of animals do not seem to constitute a priority, while electronic milking and their management systems are viewed as something quite foreign and complicated by the majority; consequently, about half of them answered that they would not be interested in learning about these systems (Fig. 2). Friedman’s statistical test was used to examine the potential existence of a statistical difference between the farmers’ views on farm management practices. According to the results, the management of dairy animals is the main interest of farmers, with a mean ranking of 7.89 (Ν = 90, χ^2^ = 370.535, df = 11, Asymp. Sig < 0.001). Principal Component Analysis (PCA) was used to examine the structure of the farmers’ views regarding knowledge and training on farm management practices and two significant factors emerged that explain 75 % of the total variance (a-Cronbach = 0.894, ΚΜΟ = 0.858, χ^2^ = 816.082, df = 66, p < 0.001).

Table 2 illustrates the loadings that emerged from the analysis, which are the partial correlation coefficients of the twelve variables with each of the two factors. The first factor, in order of significance to its formation, consists of the variables “management of replacement stock”, “management of dairy animals”, “rate of stock replacement”, “management of pregnant animals”, “improved sanitation conditions”, “lairage systems & well-being”, and “use of improved breeds”. This factor essentially expresses the desire of the farmers to acquire “knowledge on the zootechnical management” of their farms. More specifically, the management of genetic material bears the greatest significance in the formulation of this factor. The second factor, in order of significance for its formulation, consists of the variables “electronic milking systems and their management”, “management practices for organic production” and “recording, analysis and monitoring of production, animal health and reproduction”. The second factor voices the desire of the farmers to acquire “knowledge on new farm control and management systems”. Finally, the third factor comprises a desire for “knowledge on animal feed production” and includes the variables “use of rational feeding systems” and “quality, digestibility and production of animal feed”.

**Table 2:** Factor loadings regarding the farmers’ interest in knowledge and training on farm management practices

Factor loadings			
Variable	Following rotation		
Management of replacement stock	*0.915*	0.049	0.206
Management of dairy animals	*0.878*	0.158	0.207
Rate of stock replacement	*0.873*	0.011	0.265
Management of pregnant animals	*0.821*	0.125	0.262
Improved sanitation conditions	*0.783*	0.064	0.344
Lairage systems & well-being	*0.722*	0.279	0.294
Use of improved breeds	*0.681*	0.240	0.067
Electronic milking systems and their management	0.195	*0.808*	0.153
Management practices for organic production	−0.056	*0.769*	0.011
Recording analysis and monitoring of production animal health and reproduction	0.307	*0.719*	0.221
Use of rational feeding systems	0.304	0.214	*0.866*
Quality digestibility and production of animal feed	0.370	0.133	*0.856*

**Fig. 3:**
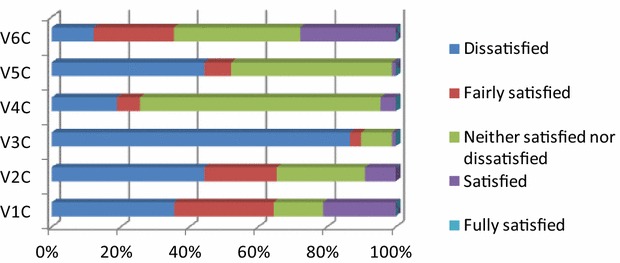
Percentage points regarding the farmers’ satisfaction with the actions of various stakeholders. V1C = local authorities; V2C = regional authorities; V3C = central government (State); V4C = scientific bodies (universities); V5C = ecological organizations and citizen groups; V6C = local press

Next, the farmers’ views were examined as to their degree of satisfaction with the attitudes of the various stakeholders involved in livestock farming (Fig. 3). The farmers express the highest level of satisfaction with the local press, which seems to portray livestock farming issues in a satisfactory manner. Only 21.1 and 8.9 % are satisfied with the actions taken by the local and regional authorities respectively, while 44.4 % are dissatisfied.

The highest level of dissatisfaction involves the central government at a rate of 86.7 %, while a high percentage is also dissatisfied with ecological organizations (44.4 %), since they consider them to be responsible for the increased number of wolves in the prefecture in recent years. As regards the scientific bodies, the majority (36.7 %) is neither satisfied nor dissatisfied with them. The main reason behind this dissatisfaction lies on the bureaucracy of public services and is also related to the state authorities responsible for the agricultural policy in Greece. Friedman’s statistical test was used to examine the potential existence of a statistical difference between the farmers’ views and their satisfaction with the actions of various stakeholders. According to the results, the farmers are mostly satisfied with the local press with a mean ranking of 4.54 (Ν = 90, χ^2^ = 139.01, df = 5, Asymp. Sig < 0.001).

After using Principal Component Analysis (PCA) to examine the structure of the farmers’ views in relation to the significance of the actions of various stakeholders two significant factors emerged. The factors explain 65.12 % of the total variance (a-Cronbach = 0.720, ΚΜΟ = 0.698, χ^2^ = 128.110, df = 15, p < 0.001) and the variables that “belong” to each factor are the ones whose loading is close to or higher than 0.5 (Table 3). The first factor, in order of significance, consists of the variables “Local Authorities”, “Regional Authorities” and “Central government (State)” and basically concerns the actions of “Public Administration”. The second factor involves “non-governmental bodies” and consists of the variables “scientific bodies (universities)”, “local press” and “ecological organizations and citizen groups”.

**Table 3:** Factor loadings of the data regarding the farmers’ satisfaction with the actions of various stakeholders

Factor loadings		
Variable	Following rotation	
Local authorities	*0.837*	0.023
Regional authorities	*0.800*	0.329
Central government (State)	*0.672*	0.041
Scientific bodies (universities)	0.061	*0.837*
Local press	0.009	*0.790*
Ecological organizations and citizen groups	0.374	*0.690*

Finally, as regards the integrated development of livestock farming, factor analysis was applied to all factors that emerged from the above-mentioned analyses. Three factors emerged that also constitute a typological presentation of the objectives, expectations and views of the farmers (Table 4). The first factor comprises the farmers’ “sustainable and economic goals” and their “desire for knowledge on zootechnics and animal feed production”. The second factor comprises the farmers’ “desire for knowledge on new farm control and management systems”, and also includes “satisfaction with the actions of non-governmental bodies”. Finally, the third factor comprises their “satisfaction with the actions of the Public Administration” and their “focus on the valorization of family labor and increased farm investments”.

**Table 4:** Factor loadings of the data regarding development factors for livestock farming

Factor loadings			
Variable	Following rotation		
Sustainable and economic goals	*0.842*	0.055	−0.132
Desire for knowledge on zootechnics	*0.732*	0.141	0.321
Desire for knowledge on animal feed production	*0.576*	−0.244	−0.455
Desire for knowledge on new farm control and management systems	0.023	*0.721*	−0.139
Non-governmental bodies	−0.021	*−0.652*	−0.056
Public administration	0.007	−0.224	*0.736*
Family labor and investment increase	0.151	0.513	*0.556*

## Discussion

The preceding analysis revealed some basic pillars of change that along with the views and perceptions of livestock farmers may lead to the increase of the sector’s competitiveness. These views can be taken into account in the application of agricultural policy measures. The results of the typology of the views and objectives expressed by sheep and goat farmers indicate that special attention should be paid to their training in the organization and management of their farms to achieve the best use of the available inputs (Rezitis et al. 2005).

Most livestock farmers have not completed elementary education, either because they have only attended primary school or only some grades of primary school; therefore, their involvement in livestock farming was the result of lacking qualifications and alternative professional options. In addition, although the population under study cannot be regarded as elderly, since approximately half are aged 35–55 years, nevertheless there is a visible absence of young people. Almost 50 % of the respondents have been involved in livestock farming for over 20 years. This specific type of farm does not appeal to young people or encourage them to become involved in livestock farming. According to sheep and goat farmers, at least two family members are employed for 10–12 h daily on the farm and their income ranges from 10,000 to 30,000 euros.

As regards the significance of livestock farming objectives, two main antecedents were highlighted. The first one is an expression of their “sustainable and economic objectives”. The second one concerns the valorization of family labor in conjunction with an increase in investments. The typological analysis shows that sheep and goat farms focus more on damage avoidance, while aiming to take advantage of family labor to improve their economic results. After examining the structure of the farmers’ views on knowledge and training regarding farm management practices, two main factors were highlighted. The first factor is an expression of the farmers’ interest in “knowledge on zootechnics”. The second factor is an expression of the farmers’ interest in “knowledge regarding new farm control and management systems”. Finally, the third factor comprises their interest in “knowledge on animal feed production”. The farmers point to the need for training on issues related to zootechnical management, with a particular focused interest in renewing the genetic material of their farms. In addition, there is a need expressed to valorize the “nutrition” input. At the same time, the farmers voice a desire to apply modern control and management systems for their farms.

The installation of automated animal feeding systems is expected to improve animal feed quality and lead to a reduction in feeding costs, while improving labor efficiency and animal management (Aggelopoulos et al. 2015). The decrease in the feeding costs can be achieved through the elaboration of a balanced and economic feeding system that will be based on the animals’ needs. Such needs are linked to the genetic breeding material and farm conditions, the nutritional content of the animal feed and the sound facilities for mixing, feeding and storing animal feed (Aggelopoulos et al. 2009). A reduction in animal feed expenses could also be reached by increasing production of at least part of the animal feed required by the farm (Milán et al. 2014). As regards the use of genetic material, it is deemed essential for farms to use the correct system in renewing their existing genetic material or in commercial crossbreeding with suitable domestic breeds, so that the relevant economic results are improved (Aggelopoulos et al. 2014). It is a fact that Greece lacks the relevant organizational structures and therefore, integrated genetic improvement programs do not exist. Consequently, producers face grave difficulties in acquiring the animals they need for reproduction, particularly female animals, at affordable prices. The planning and financing of an effective genetic improvement plan for sheep and goat farming in Greece will be a decisive step towards satisfying the producers’ need to secure the right animals for reproduction in a timely and economically beneficial manner. This can be realized through the use of suitable genetic material, with the right focus and in correlation with the local conditions for the production and trade of the products (Pavloudi et al. 2013).

The farmers’ satisfaction with stakeholders’ actions is mainly related to non-governmental bodies. Nevertheless, they express great dissatisfaction and identify major inefficiencies on behalf of the public administration. Dissatisfaction relates to strategic issues of addressing the zoonosis problem, for the fastest possible protection of livestock in the wider region, but also to the bureaucracy of government services and policy makers (Aggelopoulos et al. 2008). Livestock farmers usually stress the issues of vaccination of animals and compensations, whereas they argue that state authorities trivially confront many of their problems; already deteriorating due to the economic crisis. Public authorities can address the issue of “trust” on behalf of the farmers, through the implementation of programs that will contribute to a shift in the farmers’ attitudes and behavior towards meeting effectively current demands. The success of such a program depends on whether it will address actual needs, rather than simply the trainers’ perceptions. A change in the attitudes and behavior of policy-makers and training bodies is also required so that training takes into account the following results: the farmers interested in zootechnical training and animal feed production are those who have set ecological and economic objectives; those who are interested in acquiring knowledge on new control systems are at the same time dissatisfied with non-governmental bodies; and finally, the farmers who set social and economic objectives are satisfied with the actions of the public administration.

## Conclusion

The present study aimed to investigate the sheep and goat farmers’ perceptions and attitudes on issues related to the future growth of the sector in the Greek region of Evros. It can be argued that the study is considered significant due to the exceptional characteristics of the region, if the specific sector and its former involvement in livestock farming is adapted to modern production systems. The main objective set by sheep and goat farmers is to avoid any damage or additional cost to their farming activities. This is largely related to profit maximization rather than soil maintenance and conservation that is placed at the bottom of their list of priorities. Apparently, a major ethical question arises that in combination with the farmers’ low educational level and lack of information, results in ignorance regarding the sustainable management of natural resources. Consequently, livestock farming seems to face a grim future. As regards animal management practices, the sheep and goat farmer’s attitude towards sanitation conditions is deemed satisfactory, however they do not seem to pay sufficient attention to production, health and reproduction related issues.
